# Low-cost, high-impact altruistic punishment promotes cooperation cascades in human social networks

**DOI:** 10.1038/s41598-018-38323-7

**Published:** 2019-02-14

**Authors:** Robert M. Bond

**Affiliations:** Ohio State University, School of Communication, Columbus, OH USA

## Abstract

Theoretical models and experiments suggest that social networks may significantly impact the emergence and stability of cooperation in humans. Similarly, theoretical models and experiments have shown that punishing behavior can significantly increase cooperative behavior in individuals. However, how punishing impacts the effects of social networks on cooperation is not yet understood. Here, I examine a set of laboratory experiments in which participants choose to cooperate or defect under differing punishment arrangements. Through analysis of the experiment as a network, I evaluate how institutional arrangements affect the degree to which social networks promote cooperative behavior. The results show that cooperative behavior spreads from person-to-person in all versions of the game, but that in versions of the game with low-cost, high-impact punishment the influence both endures for more rounds and spreads further in the network. These results show that the extent to which cooperative behavior cascades is affected by the institutional arrangements that govern game play.

## Introduction

Research across disciplines has long endeavored to understand the mechanisms through which cooperation emerges and is sustained. Much of this research analyzes the behavior of the individual, and attempts to understand why an individual would bear a cost for the benefit of others in the absence of a direct benefit to the individual. Institutional arrangements, often manifested through the opportunity to punish free riders, have been proposed as mechanisms to encourage and maintain cooperation^1–3^. More recently, an area of focus has been explaining cooperative behavior through the relationships between people, including those not directly involved in the exchange. For example, those who are genetically related to one another are more likely to cooperate with one another^4,5^. This work has investigated how social networks may promote or depress levels of cooperation, depending on the dynamics within them. Indeed, theoretical models of cooperation suggest that social networks may enable cooperation to evolve and endure^6–8^. However, research has thus far not investigated how institutional arrangements interact with networks to affect cooperation.

The opportunity to punish is frequently theorized to represent an institutional choice that enables the sanctioning of free-riders^1^. Theoretical models suggest that altruistic punishment is evolutionarily stable^9^ and effective at promoting cooperation^2,3^. In one-shot experiments, altruistic punishment has been shown to significantly increase cooperative behavior^10–14^. People often pay a personal cost to punish the uncooperative behavior of others, inducing future cooperation. In one-shot interactions punishment is thought to be altruistic, as it is not possible for the future cooperation of the punished individual to directly benefit the punisher. Importantly, the effect of punishment on uncooperative behavior varies depending on the cost and impact of punishment^12^. In particular, cooperation is best maintained when punishment is low-cost enough to be used with frequency and the impact of punishment is sufficient to dissuade free-riding, but when the impact of punishment is limited it is no longer effective at dissuading uncooperative behavior. Whether the relationship between the impact of punishment on individual behavior in turn affects social network dynamics surrounding cooperation is thus far unknown.

There are various explanations for why cooperative behavior may spread in networks. For example, research has shown that when one individual acts in an altruistic way, others who view that altruism may be more likely to also behave altruistically in the future^15–17^. Similarly, research has often shown that people’s cooperative behavior is conditional on the behavior of others, in which people give more when others have done so^18,19^. Participants give more when others do so, perhaps because the social norm around giving is stronger. Theoretical models suggest that norms surrounding cooperation may emerge in small-scale groups and such norms are effective at promoting cooperation between group members^20^. These mechanisms suggest that social learning about cooperative behavior may vary depending on how behavior is impacted by the institutional arrangements that govern game play. Because the effectiveness of punishment impacts cooperation overall it may similarly impact the extent to which social norms are established and therefore the extent to which cooperative behavior spreads through the network.

One mechanism through which cooperative behavior may spread through social networks is through imitation. According to social learning theory^21^ people learn about the behavior of others through direct experience. The theory posits that when we observe the behavior of others and view it in a positive light, we may be more likely to imitate that behavior. Observation of others may affect expectations of what is an acceptable way to behave through a change in our beliefs about social norms about cooperation^19,22–24^. Importantly, a review of studies of cooperation showed that social sanctions (in behavioral economic games these are manifested through punishments from one individual to another) are crucial for norm enforcement^19^. Similarly, survey work^25^ shows that although people have varying normative views of cooperation, that in games with punishment stable contribution patterns emerge. If cooperative behavior spreads through networks in part because of the development or updating of social norms, then the institutions that govern game play, particularly concerning the costs and impacts of social sanctioning, may substantially impact the degree to which cooperation spreads from person-to-person.

In a typical behavioral public goods experiment it is clear to see how this may take place. The public goods game environment is new to most players, so although norms about cooperation in general may be familiar to a participant, how those norms may apply to the behavioral game may be uncertain. If so, observation of the game play of those a player interacts with may be influential for the development of a norm about what levels of contribution are normative in the context of the game. Specifically, in a given round of the experiment, a player may observe that one of the other participants in her group has contributed to the public good at a high level. In this scenario, she may be more likely to give at a high level in subsequent rounds of the game because she has updated her beliefs about what levels of contribution are normative. In versions of the experiment in which punishment is possible the development of norms is likely to happen more quickly. This is because social sanctioning may reinforce normative behavior – high contributors are typically not punished, but low contributors are more frequently, which reinforces a norm of acceptable levels of cooperation. In this way, institutional arrangements may impact how quickly norms are developed, which may in turn affect the degree to which the behavior of others is impactful.

Recent work has extended individual-level experiments on cooperation in the lab by investigating cooperation in social networks^26–37^. Much of this work investigates how the behavior of others in the game governs tie choice^29–34^ or is affected by existing ties^4,5,26^, and the subsequent levels of cooperation in future rounds of the game. However, this work does not investigate how the parameters of game play, such as the cost and impact of punishment, affect network dynamics related to cooperation. Because institutional arrangements are known to affect norms about cooperation, and norms are known to be one mechanism through which behaviors spread from person to person^38^, a question emerges about the extent to which institutional arrangements affect the spread of cooperation in social networks.

This study extends our understanding of cooperation in social networks in two important ways. First, the impact of punishment varies across versions of the experiments analyzed here. Although previous work has established that the impact of punishment has important consequences for the level of cooperation at the individual level^12^, it is not known whether the impact of punishment similarly affects network dynamics related to cooperation. In an individual-level analysis of the data investigated here^12^, researchers found that contributions are significantly higher in a version of the game in which punishments are low-cost and high-impact. One interpretation of this result is that social norms about cooperation are most effectively developed when the cost-to-impact ratio is low. Second, the experimental networks investigated here are composed of a more representative sample^12^ than those that have been previously analyzed for cascades of cooperation^27^, which have investigated only college students. This is important not only for generalizability, but also because the expectation that participants did not know one another or have a sense of group identity prior to the experiment is nearly certain. This is important for understanding the effect of network relationships when the likelihood of pre-existing shared norms or beliefs about the actions of others that are tied to the known attributes of other game players is low, unlike when participants are known to be students at the same university (see the Supplementary Information for more discussion of the differences between the game setup in the two sets of experiments).

To study how the impact of punishment affects social influence in cooperative behavior, I analyze data from previously published public goods experiments in which the costs and impact of punishment varied^12^. Participants were arranged into groups of three and began each round of the experiment with an endowment of 20 monetary units (MU). Participants then were tasked with deciding if they would contribute to the group project, and if so how much of their endowment to contribute (between 0 and 20 MU). The total number of MU contributed to the group project was multiplied by 1.5 and split evenly to the group members. In the version of the game with no punishment the round would end at this point. There were four versions of the game with punishment, with varying costs and impact of punishment associated with each. In the versions of the game with punishment, participants were then given the option to pay a personal cost to reduce the MU a group member has. The punishment costs and impact varied across versions of the game. In the low-cost, low-impact version, punishment cost 1 MU and reduced the income of the target by 1 MU. In the high-cost, low-impact version, punishment cost 3 MU and reduced the income of the target by 1 MU. In the low-cost, high-impact version, punishment cost 1 MU and reduced the income of the target by 3 MU. In the high-cost, high-impact version, punishment cost 3 MU and reduced the income of the target by 3 MU.

Crucially for the present study, the design of the experiments ensured that participants were strictly anonymous from one another and never played a round of the game with another participant more than once. Participants played six rounds of the experiment, each with a new group of other participants with whom they had not yet interacted. These steps are frequently taken in such experiments to distinguish cooperative behavior from other processes, such as reciprocity^15,39^ and reputation^40^. This experimental design enables the experiment to be analyzed as a network in which the group members a participant plays with may influence a player in a subsequent round^27^. That is, as players move through the rounds of the experiment they become tied to other players, both directly and indirectly, through their own interaction history and the interaction history of those they have interacted with. For example, if participant A plays with participants B and C in round 1, in the subsequent round participants B and C are the first-degree alters of participant A. If participants B and C had contributed a high amount in round 1 we might expect that participant A would be more likely to contribute a high amount in round 2, having observed the contributions of participants B and C previously. If in the second round participant A plays with participants D and E, in the next round participants B and C would be the second-degree alters of participants D and E through participant A. As the rounds progress the network builds and more distant alters (further in social network terms) may influence the behavior of their group members through their decisions in the game.

## Results

**Figure 1:**
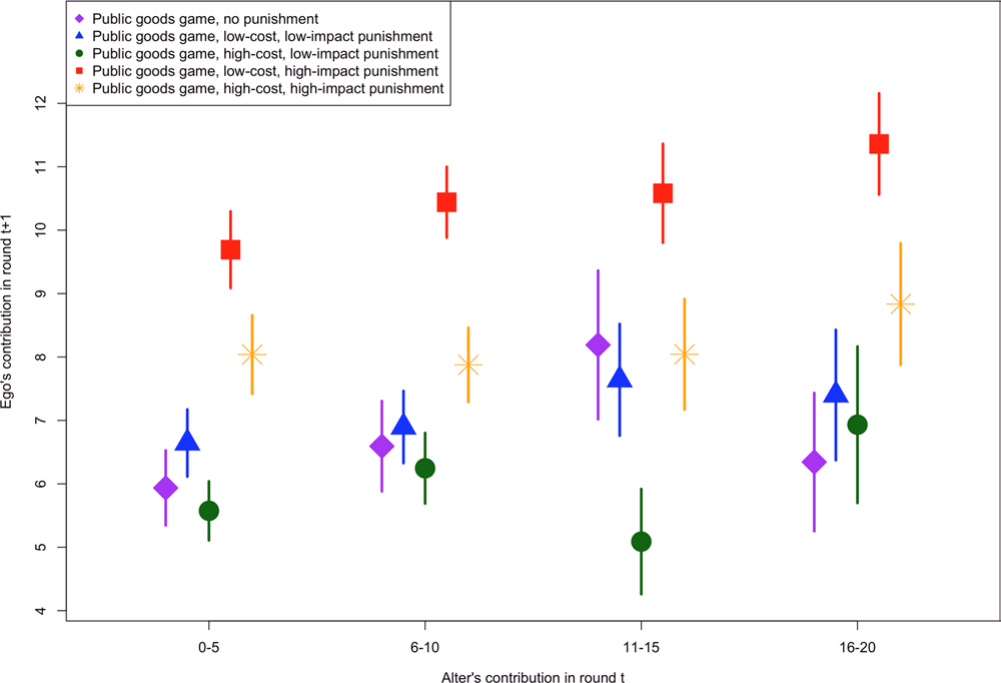
The raw relationship between the alter’s contribution in the previous round (x-axis) and the ego’s contribution in a given round (y-axis) in the Egas-Riedl public goods games. Points represent mean values and lines represent 95% confidence intervals based on the standard error of the mean.

The descriptive statistics of how player contributions are related to one another, displayed in Fig. 1, suggest that in all five versions of the game the amount a group-member (an “alter”) contributes in a given round is related to the amount the individual (the “ego”) contributes in the subsequent round. At an individual level the rate of cooperation in the low-cost, high-impact version of the game is significantly higher than in the other versions of the game, as has been analyzed elsewhere^12^. This may have important implications for network dynamics, as in this version of the game cooperation may be more likely to be viewed as normative. It is important to note that although the raw relationship is suggestive of social influence in the public goods game across versions, the raw data does not account for the fact that the contributions are constrained (participants regularly contribute the minimum or maximum amount possible given the setup of the game). The raw analysis similarly does not account for the fact that each ego and each alter play multiple rounds, meaning egos and alters are present in multiple observations both within a given round and across rounds. There is strong auto-correlation in contribution behavior, so accounting for this is important for understanding the impact of an alter’s contribution on the ego, net of the ego’s intrinsic behavior. The analyses presented below account for these factors to investigate more fully the relationship between alter and ego contributions.

To investigate how the contributions of those a focal individual is connected to through the network of previous game play impact future contributions by the focal individual a Tobit regression technique with clustered standard errors is used. This technique yields an estimate of the effect of one participant’s contribution behavior on another’s. By focusing on the ego-alter pair (rather than group-level behavior) estimates can take into account floor and ceiling effects for individual contributions (however, see the Supplementary Information for replications of the main results using group averages as the key independent variable). Further, the method helps to identify spillover effects that occur at more than one degree of separation in the network. Finally, by clustering standard errors on the ego and alter, multiple observations of both egos and alters may be accounted for (see Methods for more details).

**Figure 2:**
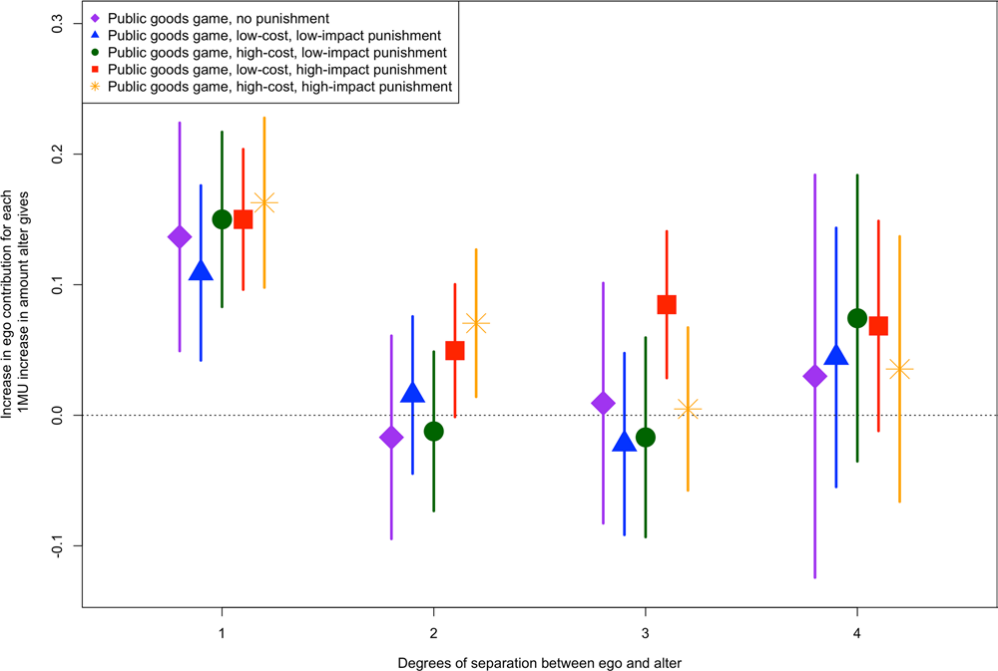
The effect of an alter’s contribution on the ego’s contribution is significant and extends up to three degrees of separation in the public goods game with low-cost, high-impact punishment, two degrees of separation in the version of the game with high-cost, high-impact punishment, and one degree of separation in versions of the game with low-impact punishment (both high- and low-cost) and no punishment. Points show average estimated effects and vertical lines represent 95% confidence intervals.

Across all five experimental versions, alters impact the contribution behavior of egos. Figure 2 visually displays the influence of an alter’s contribution on the ego’s subsequent contribution, both directly and indirectly. For example, in the version of the game without punishment, an increase of 1 MU contributed by an alter relates to an increase of 0.14 MU [95% confidence interval (CI) 0.05–0.22, *p* < 0.01] contributed by the ego in the next round. Similar effect sizes, ranging from 0.11 to 0.16 MU increases in giving by the ego were found for the four versions of the game with punishment (in all cases *p* < 0.01, see Supplementary Information Table S1). Although the focus here is on cooperative behavior, the results can likewise be interpreted as the spread of uncooperative behavior. These results suggest that as alters contribute more egos subsequently contribute more, and also that as alters contribute less egos subsequently contribute less.

In the versions of the public goods game with high-impact punishment, participants need not have directly interacted for the behavior of one individual to affect the subsequent behavior of another. In the public goods game with low-cost, high-impact punishment, an increase of 1 MU contributed by the alter’s alter relates to an increase of 0.05 MU (95% CI 0.00–0.10, *p* = 0.053) contributed by the ego two rounds later. This result is marginally significant using a two-tailed test. In the game with low-cost, high-impact punishment the effect extends to the alter’s alter’s alter (three degrees of separation) in which an increase of 1 MU contributed by the third-degree alter relates to an increase of 0.08 MU (95% CI 0.03–0.14, *p* < 0.01) contributed by the ego three rounds later. In the public goods game with high-cost, high-impact punishment, an increase of 1 MU contributed by the alter’s alter relates to an increase of 0.07 MU (95% CI 0.01–0.13, *p* < 0.05) contributed by the ego two rounds later. In both of the versions of the game with low-impact punishment and the version of the game with no punishment there is no evidence that the contributions of alters more than one degree separated from the ego impact the ego’s contribution behavior.

The effects for two and three degrees of separation in the low-cost, high-impact version of the public goods game represent the total effect of the alter’s contribution on the ego’s contribution through any pathway. For example, the effect of the third-degree alter is the result of a chain of direct interactions – the third degree alter’s behavior affects the second degree alter, the second degree alter affects the first degree alter, and finally the first degree alter affects the ego. The experiment enables a test of this by conducting a mediation analysis of the effect of the second degree alter on the ego. The results of the mediation tests (Supplementary Information Tables S9 and S10) suggest that for the public goods game with low-cost, high-impact punishment and the version of the game with high-cost, high-impact punishment, the effect of the second-degree alter’s contribution on the ego’s contribution is mediated by the first-degree alter’s contribution (note that in the other versions of the game the second-degree alter’s contribution was not significantly related to the ego’s contribution).

To test whether the relationship between alter contribution and ego contribution in the subsequent round is greater in the low-cost, high-impact punishment version of the game I analyze a model in which all five versions of the experiment are pooled and an indicator for low-cost, high-impact punishment is interacted with the alter’s contribution (Supplementary Information Tables S16–S19). For first-degree alters the interaction is not significant (B = 0.02, *p* = 0.58), indicating that first-degree alters affect ego contributions similarly whether in the low-cost, high-impact punishment version of the game or the other four versions. Similarly, for second degree alters (B = 0.03, *p* = 0.28) there is no evidence that the impact of alters in the low-cost, high-impact version of the game is different. However, for third-degree alters the interaction is positive and significant (B = 0.10, *p* < 0.01), indicating that the effect of third-degree alters is significantly higher in the low-cost, high-impact punishment version of the game than in the other four versions. For fourth-degree alters the interaction is not significant (B = 0.05, *p* = 0.34). See the Supplementary Information for more details on these models.

**Figure 3:**
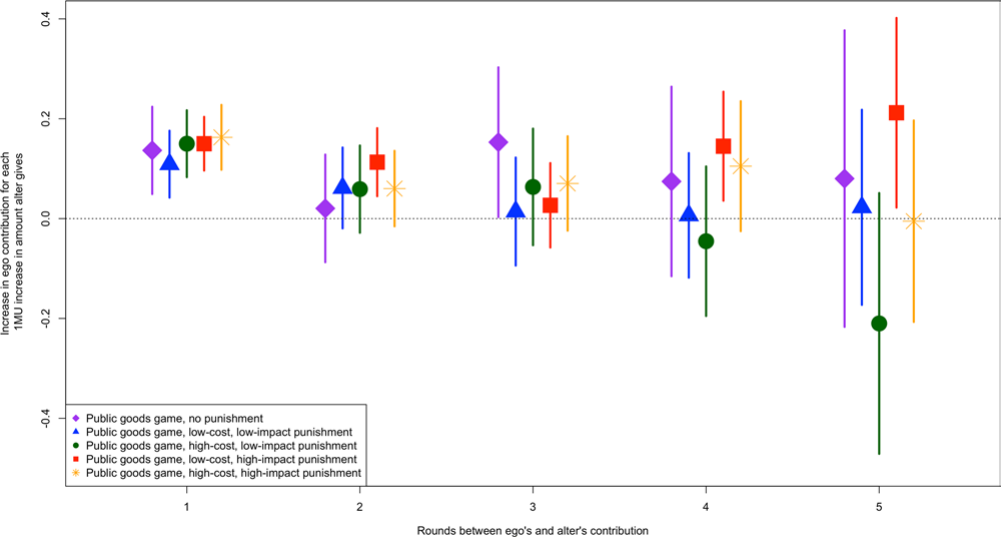
The consequence of an alter’s contribution on the ego’s contribution endures past the subsequent round of observation. In the public goods game with high-impact punishment, alter contribution has a significant effect on ego contribution for as many as four rounds later. In the public goods game with low-impact punishment, alter contribution has a significant effect on ego contribution for as many as two rounds later. In the public goods game with low-impact punishment, alter contribution has a significant effect on ego contribution for as many as three rounds later. Points show average estimated effects and vertical lines represent 95% confidence intervals.

Importantly, the effects of the contribution behavior of an alter endure over time (Fig. 3). That is, the effect of an alter may not only affect the contribution behavior of the ego in the next round of the experiment, but also in subsequent rounds. In the public goods game with low-cost, high-impact punishment, an increase of 1 MU contributed by the alter relates to an increase of 0.11 MU (95% CI 0.08–0.14, *p* < 0.01) contributed by the ego two rounds later. The effect is positive, but not significant three rounds later (*p* = 0.53). However, four rounds after the alter’s contribution, a 1 MU increase in the alter’s contribution relates to an increase of 0.14 MU (95% CI 0.09–0.19, *p* < 0.01) contributed by the ego, and five rounds after the alter’s contribution a 1 MU increase in the alter’s contribution relates to an increase of 0.21 MU (95% CI 0.11–0.31, *p* < 0.05) contributed by the ego. I note that it is curious that the effect of the alter on the ego three rounds later is not significant, but is significant both four and five rounds later. As shown in Fig. 3, the confidence interval for the effect of the alter three rounds later overlaps considerably with the confidence intervals for the effect of the alter both two and four rounds later. In the public goods game with no punishment, an increase of 1 MU contributed by the alter relates to an increase of 0.15 MU (95% CI 0.08–0.22, *p* < 0.05) contributed by the ego three rounds later, but extends no further. This effect may be spurious, as in the public goods game with no punishment the effect of the alter two rounds later is not significant (*p* = 0.73). Again, the confidence intervals for the effects of the alter on the ego in two and three rounds later overlap considerably in the public goods game with no punishment. In none of the other versions of the game did the effect of the alter on the ego significantly impact ego contributions in further than one round removed from the alter’s contribution.

To test whether the relationship between alter contribution and ego contribution in subsequent rounds is greater in the low-cost, high-impact punishment version of the game a model in which all five versions of the experiment are pooled and an indicator for low-cost, high-impact punishment is interacted with the alter’s contribution was used (Supplementary Information Tables S20–S23). For first-degree alters, the interaction is not significant in the subsequent round (B = 0.02, *p* = 0.58), two rounds later (B = 0.06, *p* = 0.12) or three rounds later (B = −0.03, *p* = 0.52), indicating that first degree alters affect ego contributions similarly in the low-cost, high-impact version of the game as in the other four versions of the game in the first three rounds after the ego and alter interact. However, alters in the low-cost, high-impact punishment version of the game have significantly more influence on the contributions of egos four rounds later (B = 0.14, *p* = 0.04) and five round later (B = 0.24, *p* = 0.04). This suggests that the impact that first degree alters have on the subsequent contributions made by egos last longer in the low-cost, high-impact version of the game than they do in the other four versions of the game. See the Supplementary Information for more details on these models.

In the versions of the experiment with punishment the punishing of an alter may have impacts on future contributions or punishing behavior by the ego. To test the relationship between alter punishments given and ego contributions, separate models for each version of the game with punishment were analyzed (Supplementary Information Table S12). In these models, only in the version of the game with high-cost, high-impact punishment was the alter’s punishment given in the previous round significantly related to ego contributions (B = 0.77, *p* < 0.01). In the other three versions of the game with punishment, alter punishments in the previous round are not significantly related to ego contributions. In none of the versions of the game was the average punishment given by alters to any other player (i.e., the average level of punishment the ego observed) in the previous round significantly related to the ego’s contribution (Supplementary Information Table S13). However, there are other pathways through which alter punishments may affect ego contributions. First, it is possible that the punishment an alter’s alter receives impacts the ego’s contribution. If so, it is likely that the punishment received by the alter’s alter would cause the alter to contribute more in the next round which may impact the ego to contribute more. There was no evidence that punishments received by the alter’s alter impacted the contribution of the ego in any of the four versions of the experiment with punishment (Supplementary Information Table S14). It is also possible that there is social influence in punishment itself, in which a participant is punished and therefore punishes more in subsequent rounds. There was no evidence that punishing behavior spread in any of the versions of the experiment (Supplementary Information Table S15).

## Discussion

As many experiments have shown, participants in the experiments examined here do not exclusively act selfishly for their own best interests. Indeed, participants regularly contribute to the public good and use punishment mechanisms to dissuade others from acting selfishly^12^. Further, the analysis presented here suggests that in all experimental versions participants observe the behavior of others and modify their own behavior depending on the level of cooperation they observe in others. Further, the results show that the extent to which they do so depends on the institutional arrangements that govern the costs and impact that altruistic punishment would have on their income and the income of others.

Previous work examining a separate set of experiments found that there were no significant differences between how cooperation spreads in games with and without punishment^27^. That research examined a smaller set of experiments and only one cost-to-impact ratio of altruistic punishment. Here, I find that in all five versions of the experiment there is evidence of social influence of cooperative behavior. This suggests that the possibility of punishment and the cost-to-impact ratio of punishment do not inhibit the social influence of cooperation across a range of punishment schemes. Importantly, I find that the punishment scheme does, in fact, impact the degree of the spread and duration of the effect of network neighbor contributions on an individual’s subsequent contributions. It is difficult to know exactly why there are differences between this study and previous work^27^ that found no difference in cooperation cascades across game conditions. There are other differences between the studies, such as the number of participants in the group, that may affect how cooperation is developed and maintained. The set of experiments examined here also include a larger number of participants so increased statistical power may enable more nuanced understanding of differences across groups.

The results presented here suggest that institutions and networks interact to affect cooperation and that cooperation is best maintained in networks in which the cost-to-impact ratio of punishment is low. This suggests that not only is individual behavior affected by the institutions that govern what types of behavior are possible and their consequences, but that the network dynamics associated with those behaviors are similarly affected by the institutions. It is important to note that the effect of punishment appears to be an institutional one – there is limited evidence that punishing behavior per se is responsible for the differences in network dynamics across experimental condition. First, I find no evidence that punishments by alters are related to subsequent punishments by egos. Further, there is limited evidence that cooperative behavior changes because of the impact or spread of punishments. In only one of the four games with punishment (high-cost, high-impact punishment) did the punishments received by the ego impact contributions in the next round. In none of the four experiments with punishment was the punishment observed by the ego in the previous round or the punishments received by an alter impactful on ego’s subsequent contributions.

These results are consistent with work that shows that in games in which only one player has the option to punish, levels of cooperation are similar to games in which all players have the option to punish^41^, that in games in which an appointed leader (“hired gun”) takes on the role of the enforcer cooperation increases^42,43^, and that cooperative behavior is increased in games in which punishment is not observed until the end of the experiment^44^. That is, the threat of punishment has been shown to increase giving in the game, regardless of actualized punishment^45^. In the experiments analyzed here, although the punishment scheme impacts the spread and duration of the social influence of cooperation, it does not appear to be the case that punishments themselves are solely responsible for these differences. The mechanism responsible for the differences in social network effects is still uncertain and future work should investigate whether punishments, norms, or some other factor accounts for differences in social influence across institutional arrangements.

This work also replicates previous findings on cascades of cooperative behavior more generally^27^, but uses a more representative sample. Previous work on cascades of cooperation has used samples of college students. Although the one-shot and anonymous nature of the experiments previously analyzed was well known, scholars have critiqued these experiments as having possibly facilitated group identity even in a one-shot, anonymous environment. Importantly, if a group identity were to account for cooperative behavior in the game, it may similarly impact the effect that network relationships would have on cooperation. Because of this, the networks analyzed here arguably embody a more strict test of the impact of social network connections on cooperation. The experiments examined here consisted of anonymous interactions over the internet and should therefore significantly reduce, if not eliminate, the problems associated with participants who may know one another. These results, therefore, provide further evidence that cascades of cooperation in behavioral games such as these are due to the behavior of the individuals participating in the experiments and how they react to the behavior of others rather than to group-level factors outside of the experiments.

These results suggest that institutional arrangements substantially govern the individual decisions and the resulting network dynamics that take place. This suggests that social sanctioning is effective not only for creating norms about cooperation at the individual level^19^, but that effective social sanctioning similarly impacts network processes related to cooperative behavior. Because of this, scholars investigating cooperation should continue to endeavor to understand how institutions impact individual decisions, but also to understand how institutional arrangements impact networks. Although there is reason to believe that the interaction of the institutions and network processes is due to differences in how norms develop depending on the rules of game play, this is not certain. Unfortunately, there is no measure of players attitudes toward what is normative about cooperation in the data analyzed here. Future work should further investigate the link between institutional arrangements, the development of social norms, and social influence surrounding cooperation. In the networks observed here the tie decisions were made by the researchers. Although this decision makes for a clean understanding of cooperation net of the effect of reputation, reputation is known to substantially impact partner choice^35^. Future work should investigate the interrelationships between institutions, individual decisions, partner choice, and network dynamics, and how they govern the emergence and stability of cooperation.

This research further demonstrates the power that social influence has on cooperative behavior even in settings in which the social ties are ephemeral. Field experiments show that the likelihood of social influence increases dramatically with tie strength^46,47^. Recent work has shown that tie strength similarly impacts cooperative behavior^36,37^. The interactions here are by their nature anonymous and short-term, yet influence still takes place. This suggests that strong relationships are not necessary for norms surrounding cooperation to emerge and influence behavior. Future work should investigate how interactions in anonymous and non-anonymous situations differ in their effect on cooperation, not only in the reputation of individuals, but also in the reputations that individuals who know one another bring to the interaction, and how these relate to social influence in the network.

## Methods

The experimental procedures used to implement the experiments for the participants evaluated in this research have been described in detail in a separate report^12^. All data analyzed in this report are secondary data with no identifying information. The experiments were explicitly designed such that no two participants would play more than one round of the experiment with one another. In such a design, the ego is connected to an alter, but participants are never re-connected to one another by two degrees of separation or fewer. Similarly, this ensures that redundant paths between participants are not possible at one and two degrees of separation, and participants are not connected to themselves through paths of two or fewer degrees. However, in later rounds of the experiment (three or more degrees of separation), these relationships are possible, so all paths that contain a self-connection or a redundant path is removed from the analysis. Further, in any instance in which there are multiple paths between an ego and alter, only the shortest path is kept.

Ego contributions are modeled using Tobit regression, which is frequently used in analyses of public goods games^16^. Tobit regression treats contributions in the game as censored when they are at the extremes (a minimum of 0 MU and maximum of 20 MU in these experiments) values. This method of analysis is preferable for censored data, as it has been shown to provide estimates that are less likely to be biased toward zero^48^. However, the resulting estimates are for a latent response variable (what the contributions of participants would be if not constrained) rather than the empirical contribution (actual contributions in the experiment).

The effect of one participant’s contribution on another participant’s contribution is estimated by including in the regression model the alter’s contribution in a previous round. For example, if estimating the ego’s contribution in round *t*, the model would include the behavior of the alter in round *t* − *k*, where *k* is the degree of separation between the ego and alter in round *t*. In this way, a direct alter would be *k* = 1, and alter’s alter would be *k* = 2, and so on. It is likely that an ego’s contribution behavior is correlated over time, so each model also includes the ego’s contribution in the same round as the round in which the alter’s behavior is being observed (i.e., *t* − *k*). To account for round effects, an indicator variable for all but one round is included in the model. To account for differences in versions of the game, an indicator variable is included for the version of the game in all models in which more than one version of the game is pooled. Huber-White sandwich errors are used, clustering on both the ego and the alter, to account for multiple observations of both each ego and each alter in the regression models. The number of participants for each version of the game is as follows: no punishment (*n* = 144), low-cost, low-impact punishment (*n* = 180), high-cost, high-impact punishment (*n* = 180), low-cost, high-impact punishment (*n* = 180), and high-cost, high-impact punishment (*n* = 162).

Finally, it is important to remember that interactions in the game were anonymous and participants were aware of this. In each round, group composition was changed randomly and each participant was aware that participants had no knowledge of each other’s behavior in previous rounds. No information about participants’ past contributions or punishment behavior was known to any other participant in a given round. As such, participants were not able to develop reputations or target one another for rewards or revenge for behavior in previous rounds.

## Supplementary information

Supplementary Information for Low-cost, high-impact altruistic punishment promotes cooperation cascades in human social networks

## Data Availability

Data are available by contacting the corresponding author of the original experiments.
