# Supplementary material for: Low-cost, high-impact altruistic punishment promotes cooperation cascades in human social networks
Source: Sci Rep. 2019 Feb 14;9:2061. doi: 10.1038/s41598-018-38323-7 (PMC6376046; doi:10.1038/s41598-018-38323-7)
Supplement: Supplementary file 1 — Supplementary Information for Low-cost, high-impact altruistic punishment promotes cooperation cascades in human social networks [file 41598_2018_38323_MOESM1_ESM.pdf]

Supplementary Information for Low-cost, high-impact altruistic punishment promotes cooperation cascades in human social networks

Robert M. Bond

November 27, 2018

1 Introduction

Table S1 shows the results of regression analysis of the effect of the first degree alter's contribution on the ego's contribution separately for the five versions of the public goods game. Table S2 shows the results of regression analysis of the effect of the second degree alter's contribution on the ego's contribution for the five versions of the public goods game. Table S3 shows the results of regression analysis of the effect of the third degree alter's contribution on the ego's contribution for the five versions of the public goods game. Table S4 shows the results of regression analysis of the effect of the fourth degree alter's contribution on the ego's contribution for the five versions of the public goods game.

Table S5 shows the results of regression analysis of the effect of the first degree alter's contribution on the ego's contribution two rounds later for the five versions of the public goods game. Table S6 shows the results of regression analysis of the effect of the first degree alter's contribution on the ego's contribution three rounds later for the five versions of the public goods game. Table S7 shows the results of regression analysis of the effect of the first degree alter's contribution on the ego's contribution four rounds later for the five versions of the public goods game. Table S8 shows the results of regression analysis of the effect of the first degree alter's contribution on the ego's contribution five rounds later for the five versions of the public goods game.

Table S9 shows the results of regression analysis of the mediation of the effect of the second degree alter's contribution on the ego's contribution by the first degree alter's contribution in the version of the public goods game with low-cost, high-impact punishment. Table S10 shows the results of regression analysis of the mediation of the effect of the second degree alter's contribution on the ego's contribution by the first degree alter's contribution in the version of the public goods game with high-cost, high-impact punishment.

Table S11 shows the results of regression analysis of the effect of the average of first degree alter's contributions on contributions made by the ego in the next round in the five versions of the public goods game.

Table S12 shows the results of regression analysis of the effect of the first degree alter's punishment given to the ego in the previous round on contributions made by the ego in the four versions of the public goods game with punishment.

Table S13 shows the results of regression analysis of the effect of the average of first degree alter's punishments given to the ego in the previous round on contributions made by the ego in the four versions of the public goods game with punishment.

Table S14 shows the results of regression analysis of the effect of the second degree alter's punishment received on contributions made by the ego two rounds later in the four versions of the public goods game with punishment.

Table S15 shows the results of regression analysis of the effect of the first degree alter's punishment received on ego punishment given in the four versions of the public goods game with punishment.

Table S16 shows the results of regression analysis of the interaction between punishment scenario and the first degree alter's contribution on ego contribution in the five versions of the public goods game. Table S17 shows the results of regression analysis of the interaction between punishment scenario and the second degree alter's contribution on ego contribution in the five versions of the public goods game. Table S18 shows the results of regression analysis of the interaction

between punishment scenario and the third degree alter's contribution on ego contribution in the five versions of the public goods game. Table S19 shows the results of regression analysis of the interaction between punishment scenario and the fourth degree alter's contribution on ego contribution in the five versions of the public goods game.

Table S20 shows the results of regression analysis of the interaction between punishment scenario and the first degree alter's contribution on ego contribution two rounds later in the five versions of the public goods game. Table S21 shows the results of regression analysis of the interaction between punishment scenario and the first degree alter's contribution on ego contribution three rounds later in the five versions of the public goods game. Table S22 shows the results of regression analysis of the interaction between punishment scenario and the first degree alter's contribution on ego contribution four rounds later in the five versions of the public goods game. Table S23 shows the results of regression analysis of the interaction between punishment scenario and the first degrees alter's contribution on ego contribution five rounds later in the five versions of the public goods game.

Although the experiments used by Fowler and Christakis (FC) [FC10] are similar to those examined here, the experimental procedures used differ from those conducted by Egas and Riedl (ER) [ER08] that are analyzed here. Of course, the most important difference is in the experimental conditions analyzed – FC [FC10] analyze only games in which there is either no punishment, or low-cost, high-impact punishment. I analyze five versions of the game, including the two analyzed by FC [FC10] and versions of the game with low-cost, low-impact punishment, high-cost, low-impact punishment, and high-cost, high-impact punishment. The sample used by the two studies differ. All of the participants in the experiments analyzed by FC [FC10] are students, while the participants in the experiments conducted by ER [ER08] that I examine may be any adult who chose to participate. Relatedly, in the experiments analyzed by FC [FC10] the participants completed the study in a lab on campus, while the participants in the experimentsconducted by ER [ER08] that I analyze could complete the study in a setting of their choosing over the internet. Finally, in the experiments analyzed by FC [FC10] the groups in each round were of size four, while in the experimentsconducted by ER [ER08] that I examine the groups were of size three.

Although the models used below cluster on the ego and alter, it is possible that session-level factors account for some of the relationship between ego and alter contribution behavior. More specifically, it is possible that (after the first round) the data within a session become dependent in ways that the modeling strategy is not accounting for. One way of accounting for such dependencies would be to include a clusters on the session as well. However, there are only nine sessions in the data, which fall well short of recommendations of roughly 30-40 clusters. To test for the degree of dependency in the data, I conducted an intraclass correlation analysis for each of the models. To do so, I created predicted values for each participant using the model. Then, I calculated the intraclass correlation on the predicted values. High intraclass correlation values would indicate that group structure, or in this case session-level differences in behavior, was likely unaccounted for in the model. However, in none of the models did I find an intraclass correlation above 0.06. These results indicate that session-level variation is unlikely to account for the relationship between ego and alter contribution.

	Public Goods Game with no punishment	Public Goods Game with low-cost, low-impact punishment	Public Goods Game with high-cost, low-impact punishment	Public Goods Game with low-cost, high-impact punishment	Public Goods Game with high-cost, high-impact punishment
Alter contribution t-1	0.14 (0.04)**	0.11 (0.03)**	0.15 (0.03)***	0.15 (0.03)***	0.16 (0.03)***
Ego contribution t-1	0.70 (0.05)***	0.69 (0.04)***	0.74 (0.04)***	0.75 (0.03)***	0.70 (0.04)***
Round 3	−0.71 (0.75)	−0.37 (0.57)	−0.52 (0.55)	−1.03 (0.48)*	−0.13 (0.58)
Round 4	−0.88 (0.82)	−1.43 (0.60)*	−0.23 (0.58)	−1.20 (0.49)*	−0.01 (0.57)
Round 5	−3.01 (0.85)***	−1.74 (0.60)**	−1.07 (0.60)	−0.46 (0.48)	−0.44 (0.60)
Round 6	−1.32 (0.86)	−1.71 (0.63)**	−1.17 (0.64)	0.21 (0.52)	−1.28 (0.61)*
Constant	0.08 (0.74)	1.07 (0.61)	−0.75 (0.56)	2.13 (0.50)***	0.87 (0.59)
AIC	7149.00	9435.75	8996.99	9807.39	8761.88
Log Likelihood	-3566.50	-4709.88	-4490.49	-4895.70	-4372.94
Num. obs.	1440	1800	1800	1800	1620
AIC	7149.00	9435.75	8996.99	9807.39	9807.39
Log Likelihood	-3566.50	-4709.88	-4490.49	-4895.70	-4895.70
Num. obs.	1440	1800	1800	1800	1800

$^{***}p < 0.001$, $^{**}p < 0.01$, $^{*}p < 0.05$

Table S1: **Tobit regression analysis of first-degree alter's contribution on ego's subsequent contribution.** Tobit regression model of alter's contribution on ego's contribution in the next round, controlling for contribution by ego in a previous round and fixed effects for each round. To account for multiple observations of egos and alters, Huber-White sandwich errors are used clustering on each ego and alter.

	Public Goods Game with no punishment	Public Goods Game with low-cost, low-impact punishment	Public Goods Game with high-cost, low-impact punishment	Public Goods Game with low-cost, high-impact punishment	Public Goods Game with high-cost, high-impact punishment
Alter's Alter's contribution t-2	-0.02 (0.04)	0.02 (0.03)	-0.01 (0.03)	0.05 (0.03)	0.07 (0.03)*
Ego contribution t-1	0.70 (0.05)***	0.64 (0.04)***	0.68 (0.04)***	0.62 (0.03)***	0.65 (0.04)***
Round 4	-0.11 (0.61)	-1.05 (0.43)*	0.23 (0.42)	-0.90 (0.36)*	0.19 (0.41)
Round 5	-2.42 (0.66)***	-1.84 (0.45)***	-0.30 (0.47)	-0.23 (0.38)	-0.27 (0.46)
Round 6	-1.51 (0.63)*	-1.88 (0.46)***	-1.00 (0.49)*	0.91 (0.42)*	-1.42 (0.44)**
Constant	-0.08 (0.67)	1.51 (0.53)**	-0.47 (0.53)	4.14 (0.45)***	1.76 (0.50)***
AIC	11134.15	14993.28	14246.63	16095.46	14168.68
Log Likelihood	-5560.08	-7489.64	-7116.32	-8040.73	-7077.34
Num. obs.	2304	2880	2880	2880	2592

$^{***}p < 0.001$, $^{**}p < 0.01$, $^{*}p < 0.05$

Table S2: **Tobit regression of second-degree alter's contribution on ego's subsequent contribution.** Tobit regression model of alter's alter's contribution on ego's contribution two rounds later, controlling for contribution by ego in two rounds previous and fixed effects for each round. To account for multiple observations of egos and alters, Huber-White sandwich errors are used clustering on each ego and alter.

	Public Goods Game with no punishment	Public Goods Game with low-cost, low-impact punishment	Public Goods Game with high-cost, low-impact punishment	Public Goods Game with low-cost, high-impact punishment	Public Goods Game with high-cost, high-impact punishment
Alter's Alter's Alter's contribution t-3	0.01 (0.05)	−0.02 (0.03)	−0.02 (0.04)	0.08 (0.03)**	0.00 (0.03)
Ego's contribution t-3	0.60 (0.06)***	0.60 (0.04)***	0.69 (0.05)***	0.65 (0.03)***	0.52 (0.04)***
Round 5	−2.57 (0.69)***	−1.06 (0.47)*	−0.47 (0.48)	−0.06 (0.36)	−0.33 (0.44)
Round 6	−1.12 (0.66)	−0.92 (0.47)*	−0.76 (0.50)	0.88 (0.40)*	−1.37 (0.43)**
Constant	−0.16 (0.78)	0.73 (0.60)	−1.20 (0.63)	3.25 (0.45)***	3.44 (0.54)***
AIC	9964.12	13529.36	12685.24	14664.68	13166.43
Log Likelihood	-4976.06	-6758.68	-6336.62	-7326.34	-6577.22
Num. obs.	2112	2640	2640	2640	2376

$^{***}p < 0.001$, $^{**}p < 0.01$, $^{*}p < 0.05$

Table S3: **Tobit regression of third-degree alter's contribution on ego's subsequent contribution.** Tobit regression model of alter's alter's alter's contribution on ego's contribution three rounds later, controlling for contribution by ego in three rounds previous and fixed effects for each round. To account for multiple observations of egos and alters, Huber-White sandwich errors are used clustering on each ego and alter.

	Public Goods Game with no punishment	Public Goods Game with low-cost, low-impact punishment	Public Goods Game with high-cost, low-impact punishment	Public Goods Game with low-cost, high-impact punishment	Public Goods Game with high-cost, high-impact punishment
Alter's Alter's Alter's Alter's contribution t-4	0.03 (0.08)	0.04 (0.05)	0.07 (0.05)	0.07 (0.04)	0.04 (0.05)
Ego's contribution t-4	0.40 (0.08)***	0.57 (0.06)***	0.67 (0.07)***	0.60 (0.05)***	0.34 (0.06)***
Round 6	1.39 (0.82)	−0.40 (0.51)	0.16 (0.57)	0.59 (0.41)	−1.18 (0.51)*
Constant	−2.02 (1.16)	−0.80 (0.79)	−3.19 (0.80)***	4.51 (0.59)***	4.24 (0.77)***
AIC	5033.52	6968.35	6474.44	7680.98	6898.05
Log Likelihood	-2511.76	-3479.17	-3232.22	-3835.49	-3444.02
Num. obs.	1104	1380	1380	1380	1242

$^{***}p < 0.001$, $^{**}p < 0.01$, $^{*}p < 0.05$

Table S4: **Tobit regression of fourth-degree alter's contribution on ego's subsequent contribution.** Tobit regression model of alter's alter's alter's alter's contribution on ego's contribution four rounds later, controlling for contribution by ego in four rounds previous and fixed effects for each round. To account for multiple observations of egos and alters, Huber-White sandwich errors are used clustering on each ego and alter.

	Public Goods Game with no punishment	Public Goods Game with low-cost, low-impact punishment	Public Goods Game with high-cost, low-impact punishment	Public Goods Game with low-cost, high-impact punishment	Public Goods Game with high-cost, high-impact punishment
Alter's contribution t-2	0.02 (0.05)	0.06 (0.04)	0.06 (0.04)	0.11 (0.03)***	0.06 (0.04)
Ego's contribution t-2	0.70 (0.06)***	0.64 (0.05)***	0.68 (0.05)***	0.62 (0.04)***	0.65 (0.05)***
Round 3	−0.08 (0.92)	−1.02 (0.66)	0.31 (0.65)	−0.95 (0.55)	0.18 (0.64)
Round 4	−2.37 (0.93)*	−1.80 (0.64)**	−0.15 (0.66)	−0.27 (0.54)	−0.28 (0.65)
Round 5	−1.44 (0.95)	−1.79 (0.68)**	−0.84 (0.70)	0.88 (0.61)	−1.42 (0.65)*
Constant	−0.39 (0.91)	1.08 (0.71)	−1.08 (0.71)	3.55 (0.61)***	1.84 (0.69)**
AIC	5574.03	7501.33	7128.48	8045.40	7092.28
Log Likelihood	-2780.02	-3743.66	-3557.24	-4015.70	-3539.14
Num. obs.	1152	1440	1440	1440	1296

$^{***}p < 0.001$, $^{**}p < 0.01$, $^{*}p < 0.05$

Table S5: **Tobit regression of first-degree alter's contribution on ego's contribution two rounds later.** Tobit regression model of alter's contribution on ego's contribution two rounds later, controlling for contribution by ego in two rounds previous and fixed effects for each round. To account for multiple observations of egos and alters, Huber-White sandwich errors are used clustering on each ego and alter.

	Public Goods Game with no punishment	Public Goods Game with low-cost, low-impact punishment	Public Goods Game with high-cost, low-impact punishment	Public Goods Game with low-cost, high-impact punishment	Public Goods Game with high-cost, high-impact punishment
Alter's contribution t-3	0.15 (0.07)*	0.01 (0.05)	0.06 (0.06)	0.03 (0.04)	0.07 (0.05)
Ego's contribution t-3	0.61 (0.09)***	0.61 (0.06)***	0.69 (0.07)***	0.64 (0.05)***	0.53 (0.06)***
Round 3	−2.22 (1.08)*	−0.73 (0.74)	−0.34 (0.74)	−0.01 (0.56)	−0.36 (0.70)
Round 4	−1.08 (1.08)	−1.15 (0.75)	−0.64 (0.79)	1.10 (0.64)	−1.53 (0.68)*
Constant	−1.56 (1.17)	0.31 (0.93)	−1.82 (0.88)*	3.91 (0.69)***	2.78 (0.77)***
AIC	4072.53	5555.02	5227.14	6029.40	5381.48
Log Likelihood	-2030.27	-2771.51	-2607.57	-3008.70	-2684.74
Num. obs.	864	1080	1080	1080	972

$^{***}p < 0.001$, $^{**}p < 0.01$, $^{*}p < 0.05$

Table S6: **Tobit regression of first-degree alter's contribution on ego's contribution three rounds later.** Tobit regression model of alter's contribution on ego's contribution three rounds later, controlling for contribution by ego in three rounds previous and fixed effects for each round. To account for multiple observations of egos and alters, Huber-White sandwich errors are used clustering on each ego and alter.

	Public Goods Game with no punishment	Public Goods Game with low-cost, low-impact punishment	Public Goods Game with high-cost, low-impact punishment	Public Goods Game with low-cost, high-impact punishment	Public Goods Game with high-cost, high-impact punishment
Alter's contribution t-4	0.07 (0.09)	0.01 (0.06)	−0.05 (0.07)	0.14 (0.05)**	0.11 (0.07)
Ego's contribution t-4	0.40 (0.11)***	0.53 (0.08)***	0.63 (0.08)***	0.58 (0.06)***	0.35 (0.08)***
Round 3	1.13 (1.17)	−0.31 (0.75)	−0.37 (0.84)	0.35 (0.62)	−1.17 (0.75)
Constant	−2.02 (1.50)	−0.01 (1.04)	−1.65 (1.06)	3.95 (0.81)***	3.52 (1.02)***
AIC	2668.42	3676.95	3402.59	4021.38	3602.95
Log Likelihood	-1329.21	-1833.48	-1696.29	-2005.69	-1796.47
Num. obs.	576	720	720	720	648

$^{***}p < 0.001$, $^{**}p < 0.01$, $^{*}p < 0.05$

Table S7: **Tobit regression of first-degree alter's contribution on ego's contribution four rounds later.** Tobit regression model of alter's contribution on ego's contribution four rounds later, controlling for contribution by ego in four rounds previous and fixed effects for each round. To account for multiple observations of egos and alters, Huber-White sandwich errors are used clustering on each ego and alter.

	Public Goods Game with no punishment	Public Goods Game with low-cost, low-impact punishment	Public Goods Game with high-cost, low-impact punishment	Public Goods Game with low-cost, high-impact punishment	Public Goods Game with high-cost, high-impact punishment
Alter's contribution t-5	0.08 (0.15)	0.02 (0.10)	-0.21 (0.13)	0.21 (0.10)*	-0.01 (0.10)
Ego's contribution t-5	0.37 (0.17)*	0.43 (0.12)***	0.63 (0.14)***	0.38 (0.11)***	0.40 (0.12)***
Constant	-1.23 (1.97)	0.12 (1.37)	-1.78 (1.60)	6.36 (1.43)***	2.69 (1.40)
AIC	1336.43	1827.03	1640.86	1989.80	1783.48
Log Likelihood	-664.21	-909.52	-816.43	-990.90	-887.74
Num. obs.	288	360	360	360	324

$^{***}p < 0.001$, $^{**}p < 0.01$, $^{*}p < 0.05$

Table S8: **Tobit regression of first-degree alter's contribution on ego's contribution five rounds later.** Tobit regression model of alter's contribution on ego's contribution five rounds later, controlling for contribution by ego in five rounds previous. To account for multiple observations of egos and alters, Huber-White sandwich errors are used clustering on each ego and alter.

Public Goods Game with Low-Cost, High-Impact Punishment			
	Ego's Contribution in Round t	Alter's Contribution in Round t-1	Ego's Contribution in Round t
Alter's alter's contribution t-2	0.05 (0.03)	0.19 (0.04)***	0.03 (0.02)
Alter's contribution t-1			0.14 (0.03)***
Ego's contribution t-2	0.62 (0.04)***	-0.05 (0.02)*	0.62 (0.04)***
Round 4	-0.90 (0.56)	-0.36 (0.58)	-0.88 (0.55)
Round 5	-0.23 (0.54)	-0.66 (0.59)	-0.17 (0.53)
Round 6	0.91 (0.62)	-0.01 (0.57)	0.90 (0.62)
Constant	4.14 (0.56)***	9.32 (0.56)***	2.81 (0.63)***
AIC	16095.46	16876.81	16059.41
Log Likelihood	-8040.73	-8431.41	-8021.71
Num. obs.	2880	2880	2880

$^{***}p < 0.001$, $^{**}p < 0.01$, $^{*}p < 0.05$

Table S9: **Tobit regression mediation analysis for the public goods game with low-cost, high-impact punishment.** Tobit regression models of ego and alter contributions, using Huber-White sandwich errors to account for multiple observations of egos and alters.

Public Goods Game with High-Cost, High-Impact Punishment			
	Ego's Contribution in Round t	Alter's Contribution in Round t-1	Ego's Contribution in Round t
Alter's alter's contribution t-2	0.07 (0.03)**	0.18 (0.04)***	0.04 (0.03)
Alter's contribution t-1			0.19 (0.04)***
Ego's contribution t-2	0.65 (0.05)***	0.02 (0.03)	0.65 (0.05)***
Round 4	0.19 (0.64)	-0.33 (0.67)	0.23 (0.62)
Round 5	-0.27 (0.65)	-0.41 (0.68)	-0.19 (0.65)
Round 6	-1.42 (0.65)*	-0.92 (0.71)	-1.27 (0.64)*
Constant	1.76 (0.64)**	6.96 (0.69)***	0.36 (0.71)
AIC	14168.68	14808.37	14117.98
Log Likelihood	-7077.34	-7397.19	-7050.99
Num. obs.	2592	2592	2592

$^{***}p < 0.001$, $^{**}p < 0.01$, $^{*}p < 0.05$

Table S10: **Tobit regression mediation analysis for the public goods game with high-cost, high-impact punishment.** Tobit regression models of ego and alter contributions, using Huber-White sandwich errors to account for multiple observations of egos and alters.

	DV: Ego contribution in round t				
	Public Goods Game with no punishment	Public Goods Game with low-cost, low-impact punishment	Public Goods Game with high-cost, low-impact punishment	Public Goods Game with low-cost, high-impact punishment	Public Goods Game with high-cost, high-impact punishment
Average alter contribution in Round t-1	$0.26\ (0.09)^{**}$	$0.23\ (0.07)^{**}$	$0.31\ (0.07)^{***}$	$0.30\ (0.06)^{***}$	$0.32\ (0.07)^{***}$
Ego's contribution in Round t-2	$0.69\ (0.09)^{***}$	$0.69\ (0.08)^{***}$	$0.74\ (0.07)^{***}$	$0.75\ (0.06)^{***}$	$0.70\ (0.08)^{***}$
Round 3	-0.62 (1.14)	-0.30 (0.89)	-0.35 (0.81)	-1.15 (0.69)	-0.08 (0.88)
Round 4	-0.71 (1.06)	-1.30 (0.80)	0.09 (0.75)	$-1.31\ (0.62)^{*}$	0.08 (0.70)
Round 5	$-2.81\ (1.13)^{*}$	-1.52 (0.84)	-0.70 (0.78)	-0.55 (0.59)	-0.31 (0.79)
Round 6	-0.96 (1.19)	-1.40 (0.88)	-0.69 (0.82)	0.05 (0.70)	-1.09 (0.86)
Constant	-0.95 (1.26)	-0.04 (1.18)	$-2.11\ (0.99)^{*}$	0.71 (0.86)	-0.52 (0.95)
AIC	3577.67	4719.48	4495.70	4895.27	4375.78
Log Likelihood	-1780.83	-2351.74	-2239.85	-2439.63	-2179.89
Num. obs.	720	900	900	900	810

$^{***}p < 0.001$, $^{**}p < 0.01$, $^{*}p < 0.05$

Table S11: **Tobit regression analysis of average of first-degree alter's contributions on ego's subsequent contribution.** Tobit regression model of average of alter's contributions on ego's contribution in the next round, controlling for contribution by ego in a previous round and fixed effects for each round. To account for multiple observations of egos, Huber-White sandwich errors are used clustering on each ego.

	DV: Ego contribution in round t			
	Public Goods Game with low-cost, low-impact punishment	Public Goods Game with high-cost, low-impact punishment	Public Goods Game with low-cost, high-impact punishment	Public Goods Game with high-cost, high-impact punishment
Alter's contribution in Round t-1	0.13 (0.04)***	0.17 (0.03)***	0.16 (0.03)***	0.19 (0.03)***
Ego's contribution in Round t-1	0.68 (0.04)***	0.73 (0.04)***	0.75 (0.03)***	0.69 (0.04)***
Alter's punishment of Ego in Round t-1	0.16 (0.12)	0.27 (0.23)	0.09 (0.09)	0.77 (0.23)***
Total punishment sent by Alter in Round t-1	−0.07 (0.10)	−0.40 (0.20)*	0.04 (0.09)	0.03 (0.18)
Round 3	−0.35 (0.57)	−0.54 (0.55)	−1.05 (0.47)*	−0.09 (0.58)
Round 4	−1.40 (0.61)*	−0.23 (0.58)	−1.24 (0.50)*	0.03 (0.57)
Round 5	−1.69 (0.60)**	−1.04 (0.60)	−0.48 (0.48)	−0.35 (0.60)
Round 6	−1.69 (0.63)**	−1.18 (0.64)	0.19 (0.52)	−1.15 (0.61)
Constant	0.91 (0.63)	−0.80 (0.57)	1.94 (0.52)***	0.44 (0.59)
AIC	9436.86	8997.28	9810.06	8754.11
Log Likelihood	-4708.43	-4488.64	-4895.03	-4367.06
Num. obs.	1800	1800	1800	1620

$^{***}p < 0.001$, $^{**}p < 0.01$, $^{*}p < 0.05$

Table S12: **Tobit regression of first-degree alter's punishment of ego on ego's contribution in the subsequent round.** Tobit regression model of alter's punishment given to ego on ego's contribution in the subsequent round, controlling for ego's and alter's contributions in the previous round, and fixed effects for each round. To account for multiple observations of egos and alters, Huber-White sandwich errors are used clustering on each ego and alter.

	DV: Ego contribution in round t			
Average alter contribution in Round t-1	0.25 (0.07)***	0.33 (0.07)***	0.30 (0.06)***	0.34 (0.07)***
Ego's contribution in Round t-1	0.68 (0.08)***	0.73 (0.07)***	0.77 (0.06)***	0.67 (0.09)***
Average Alter's punishments of Ego in Round t-1	0.30 (0.26)	0.41 (0.43)	−0.01 (0.18)	1.37 (0.59)*
Average total punishment sent by Alters in Round t-1	−0.17 (0.22)	−0.82 (0.43)	0.24 (0.19)	−0.09 (0.37)
Round 3	−0.26 (0.89)	−0.40 (0.81)	−1.19 (0.69)	−0.05 (0.89)
Round 4	−1.26 (0.80)	0.06 (0.74)	−1.39 (0.65)*	0.12 (0.70)
Round 5	−1.44 (0.84)	−0.67 (0.77)	−0.61 (0.60)	−0.17 (0.79)
Round 6	−1.37 (0.88)	−0.73 (0.81)	0.01 (0.70)	−0.89 (0.86)
Constant	−0.20 (1.23)	−2.09 (0.98)*	0.40 (0.89)	−1.05 (0.98)
AIC	4721.42	4496.96	4897.20	4371.01
Log Likelihood	-2350.71	-2238.48	-2438.60	-2175.51
Num. obs.	900	900	900	810

$^{***}p < 0.001$, $^{**}p < 0.01$, $^{*}p < 0.05$

Table S13: **Tobit regression of average of first-degree alter's punishments of ego on ego's contribution in the subsequent round.** Tobit regression model of average of alter's punishments given to ego on ego's contribution in the subsequent round, controlling for ego's and average of alter's contributions in the previous round, and fixed effects for each round. To account for multiple observations of egos, Huber-White sandwich errors are used clustering on each ego.

	DV: Ego contribution in round t			
	Public Goods Game with low-cost, low-impact punishment	Public Goods Game with high-cost, low-impact punishment	Public Goods Game with low-cost, high-impact punishment	Public Goods Game with high-cost, high-impact punishment
Punishment received by Alter in Round t-2	-0.12 (0.09)	-0.19 (0.26)	0.03 (0.08)	0.04 (0.15)
Alter's contribution in Round t-2	0.03 (0.04)	0.05 (0.04)	0.02 (0.04)	0.08 (0.04)
Punishment received by Ego in Round t-2	-0.12 (0.10)	-0.11 (0.20)	-0.34 (0.07)***	-0.21 (0.23)
Ego's contribution in Round t-2	0.66 (0.05)***	0.68 (0.05)***	0.69 (0.04)***	0.67 (0.05)***
Round 4	-1.08 (0.66)	0.31 (0.65)	-1.02 (0.56)	0.24 (0.64)
Round 5	-1.82 (0.64)**	-0.18 (0.67)	-0.44 (0.54)	-0.21 (0.65)
Round 6	-1.92 (0.69)**	-0.88 (0.71)	0.79 (0.62)	-1.36 (0.65)*
Constant	0.91 (0.79)	-1.12 (0.74)	3.09 (0.71)***	1.40 (0.77)
AIC	7503.53	7132.47	8038.14	7092.88
Log Likelihood	-3742.76	-3557.24	-4010.07	-3537.44
Num. obs.	1440	1440	1440	1296

$^{***}p < 0.001$, $^{**}p < 0.01$, $^{*}p < 0.05$

Table S14: **Tobit regression of second-degree alter's punishment received on ego's contribution two rounds later.** Tobit regression model of alter's alter's punishment received on ego's contribution two rounds later, controlling for ego's punishment received in the prior round, ego's and alter's contributions two rounds previous, and fixed effects for each round. To account for multiple observations of egos and alters, Huber-White sandwich errors are used clustering on each ego and alter.

	DV: Punishment by Ego in Round t			
	Public Goods Game with low-cost, low-impact punishment	Public Goods Game with high-cost, low-impact punishment	Public Goods Game with low-cost, high-impact punishment	Public Goods Game with high-cost, high-impact punishment
Alter's punishment of Ego in Round t-1	-0.01 (0.06)	0.37 (0.19)	-0.01 (0.04)	-0.02 (0.08)
Ego's punishment t-1	0.64 (0.08)***	0.72 (0.13)***	0.49 (0.06)***	0.74 (0.12)***
Round 2	0.38 (0.51)	-0.14 (0.60)	0.07 (0.34)	-0.51 (0.38)
Round 3	-0.63 (0.53)	-0.43 (0.60)	0.34 (0.30)	-0.46 (0.35)
Round 4	-0.04 (0.56)	0.23 (0.63)	0.15 (0.34)	-0.52 (0.33)
Round 5	0.40 (0.54)	0.06 (0.61)	-0.03 (0.34)	-0.41 (0.37)
Constant	-3.34 (0.52)***	-4.59 (0.70)***	-1.18 (0.27)***	-2.09 (0.32)***
AIC	2924.03	1191.54	4077.97	1848.97
Log Likelihood	-1454.02	-587.77	-2030.98	-916.49
Num. obs.	1125	1002	1277	989

$^{***}p < 0.001$, $^{**}p < 0.01$, $^{*}p < 0.05$

Table S15: **Tobit regression analysis of alter's punishment given on ego's punishment given.** Tobit regression model of alter's punishment given on ego's punishment given in the next round, controlling for contribution by ego the previous round and fixed effects for each round. To account for multiple observations of egos and alters, Huber-White sandwich errors are used clustering on each ego and alter.

	DV: Ego contribution in round t	
	Model A	Model B
Alter's contribution t-1	0.14 (0.02)***	0.13 (0.04)**
Ego's contribution t-1	0.72 (0.02)***	0.72 (0.02)***
Low-cost, high-impact punishment	2.86 (0.43)***	2.77 (0.51)***
Low-cost, low-impact punishment	0.44 (0.32)	0.54 (0.52)
High-cost, low-impact punishment	−0.26 (0.32)	−0.42 (0.50)
High-cost, high-impact punishment	1.43 (0.32)***	1.11 (0.54)*
Alter's contribution t-1 × Low-cost, high-impact punishment	0.02 (0.03)	0.03 (0.05)
Alter's contribution t-1 × High-cost, high-impact punishment		0.04 (0.05)
Alter's contribution t-1 × Low-cost, low-impact punishment		−0.01 (0.05)
Alter's contribution t-1 × High-cost, low-impact punishment		0.02 (0.05)
Round 3	−0.52 (0.26)*	−0.52 (0.26)*
Round 4	−0.72 (0.27)**	−0.72 (0.27)**
Round 5	−1.18 (0.27)***	−1.18 (0.27)***
Round 6	−0.93 (0.28)***	−0.94 (0.28)***
Constant	−0.19 (0.34)	−0.10 (0.43)
AIC	44277.56	44282.09
Log Likelihood	-22125.78	-22125.04
Num. obs.	8460	8460

$^{***}p < 0.001$, $^{**}p < 0.01$, $^{*}p < 0.05$

Table S16: **Tobit regression analysis of the interaction between punishment scenario and first-degree alter's contribution for the public goods game.** Tobit regression models of alter's contribution on ego contribution interacted with low-cost, high-impact punishment condition (Model A) or interacted with all punishment scenarios separately (Model B), controlling for ego's and alter's contributions in the previous round, fixed effects for round and for cost and impact of punishment, using Huber-White sandwich errors to account for multiple observations of egos and alters. Data in these models are the pooled data across the five experimental procedures (no punishment, low-cost and low-impact punishment, low-cost and high-impact punishment, high-cost and low-impact punishment, high-cost and high-impact punishment).

	DV: Ego contribution in round t	
	Model A	Model B
Alter's alter's contribution t-2	0.02 (0.02)	−0.01 (0.03)
Ego's contribution t-2	0.65 (0.02)***	0.65 (0.02)***
Low-cost, high-impact punishment	4.21 (0.40)***	4.00 (0.47)***
Low-cost, low-impact punishment	0.67 (0.30)*	0.44 (0.47)
High-cost, low-impact punishment	−0.31 (0.30)	−0.30 (0.45)
High-cost, high-impact punishment	2.25 (0.30)***	1.55 (0.48)**
Alter's alter's contribution t-2 × Low-cost, high-impact punishment	0.03 (0.03)	0.06 (0.04)
Alter's alter's contribution t-2 × High-cost, high-impact punishment		0.08 (0.05)
Alter's alter's contribution t-2 × Low-cost, low-impact punishment		0.03 (0.05)
Alter's alter's contribution t-2 × High-cost, low-impact punishment		−0.00 (0.05)
Round 4	−0.36 (0.20)	−0.36 (0.20)
Round 5	−0.89 (0.21)***	−0.90 (0.21)***
Round 6	−0.88 (0.21)***	−0.90 (0.21)***
Constant	0.10 (0.32)	0.32 (0.40)
AIC	70842.73	70843.01
Log Likelihood	-35409.36	-35406.50
Num. obs.	13536	13536

$^{***}p < 0.001$, $^{**}p < 0.01$, $^{*}p < 0.05$

Table S17: **Tobit regression analysis of the interaction between punishment scenario and second-degree alter's contribution for the public goods game.** Tobit regression models of alter's alter's contribution on ego contribution interacted with low-cost, high-impact punishment condition (Model A) or interacted with all punishment scenarios separately (Model B), controlling for ego's and alter's contributions in two rounds previous, fixed effects for round and for cost and impact of punishment, using Huber-White sandwich errors to account for multiple observations of egos and alters. Data in these models are the pooled data across the five experimental procedures (no punishment, low-cost and low-impact punishment, low-cost and high-impact punishment, high-cost and low-impact punishment, high-cost and high-impact punishment).

	DV: Ego contribution in round t	
	Model A	Model B
Alter's alter's alter's contribution t-3	−0.00 (0.02)	0.01 (0.04)
Ego's contribution t-3	0.61 (0.02)***	0.61 (0.02)***
Low-cost, high-impact punishment	4.67 (0.46)***	4.78 (0.54)***
Low-cost, low-impact punishment	0.60 (0.34)	0.84 (0.56)
High-cost, low-impact punishment	−0.22 (0.34)	−0.03 (0.54)
High-cost, high-impact punishment	2.91 (0.34)***	2.90 (0.55)***
Alter's alter's alter's contribution t-3 × Low-cost, high-impact punishment	0.10 (0.04)**	0.09 (0.05)
Alter's alter's alter's contribution t-3 × High-cost, high-impact punishment		−0.00 (0.05)
Alter's alter's alter's contribution t-3 × Low-cost, low-impact punishment		−0.03 (0.05)
Alter's alter's alter's contribution t-3 × High-cost, low-impact punishment		−0.02 (0.05)
Round 5	−0.74 (0.21)***	−0.74 (0.21)***
Round 6	−0.57 (0.21)**	−0.58 (0.21)**
Constant	−0.32 (0.36)	−0.43 (0.46)
AIC	64251.82	64257.15
Log Likelihood	-32114.91	-32114.58
Num. obs.	12408	12408

$^{***}p < 0.001$, $^{**}p < 0.01$, $^{*}p < 0.05$

Table S18: **Tobit regression analysis of the interaction between punishment scenario and third-degree alter's contribution for the public goods game.** Tobit regression models of alter's alter's alter's contribution on ego contribution interacted with low-cost, high-impact punishment condition (Model A) or interacted with all punishment scenarios separately (Model B), controlling for ego's and alter's contributions in three rounds previous, fixed effects for round and for cost and impact of punishment, using Huber-White sandwich errors to account for multiple observations of egos and alters. Data in these models are the pooled data across the five experimental procedures (no punishment, low-cost and low-impact punishment, low-cost and high-impact punishment, high-cost and low-impact punishment, high-cost and high-impact punishment).

	DV: Ego contribution in round t	
	Model A	Model B
Alter's alter's alter's alter's contribution t-4	0.04 (0.03)	0.02 (0.07)
Ego's contribution t-4	0.52 (0.03)***	0.52 (0.03)***
Low-cost, high-impact punishment	6.94 (0.66)***	6.78 (0.81)***
Low-cost, low-impact punishment	0.81 (0.49)	0.65 (0.84)
High-cost, low-impact punishment	−0.03 (0.50)	−0.40 (0.84)
High-cost, high-impact punishment	3.44 (0.49)***	3.34 (0.86)***
Alter's alter's alter's alter's contribution t-4× Low-cost, high-impact punishment	0.05 (0.05)	0.07 (0.08)
Alter's alter's alter's alter's contribution t-4× High-cost, high-impact punishment		0.01 (0.08)
Alter's alter's alter's alter's contribution t-4× Low-cost, low-impact punishment		0.02 (0.08)
Alter's alter's alter's alter's contribution t-4× High-cost, low-impact punishment		0.05 (0.08)
Round 6	0.05 (0.24)	0.05 (0.24)
Constant	−1.45 (0.51)**	−1.29 (0.70)
AIC	33223.59	33229.19
Log Likelihood	-16601.80	-16601.59
Num. obs.	6486	6486

***$p < 0.001$, **$p < 0.01$, *$p < 0.05$

Table S19: **Tobit regression analysis of the interaction between punishment scenario and fourth-degree alter's contribution for the public goods game.** Tobit regression models of alter's alter's alter's alter's contribution on ego contribution interacted with low-cost, high-impact punishment condition (Model A) or interacted with all punishment scenarios separately (Model B), controlling for ego's and alter's contributions in four rounds previous, fixed effects for round and for cost and impact of punishment, using Huber-White sandwich errors to account for multiple observations of egos and alters. Data in these models are the pooled data across the five experimental procedures (no punishment, low-cost and low-impact punishment, low-cost and high-impact punishment, high-cost and low-impact punishment, high-cost and high-impact punishment).

	DV: Ego contribution in round t	
	Model A	Model B
Alter's contribution t-2	0.05 (0.02)*	0.03 (0.05)
Ego's contribution t-2	0.65 (0.02)***	0.65 (0.02)***
Low-cost, high-impact punishment	3.82 (0.54)***	3.60 (0.64)***
High-cost, high-impact punishment	2.21 (0.38)***	1.91 (0.65)**
High-cost, low-impact punishment	−0.29 (0.39)	−0.52 (0.63)
Low-cost, low-impact punishment	0.65 (0.39)	0.32 (0.64)
Alter's contribution t-2× Low-cost, high-impact punishment	0.06 (0.04)	0.09 (0.06)
Alter's contribution t-2× High-cost, high-impact punishment		0.04 (0.06)
Alter's contribution t-2× High-cost, low-impact punishment		0.03 (0.06)
Alter's contribution t-2× Low-cost, low-impact punishment		0.04 (0.06)
Round 3	−0.35 (0.30)	−0.35 (0.30)
Round 4	−0.87 (0.30)**	−0.87 (0.30)**
Round 5	−0.84 (0.31)**	−0.85 (0.31)**
Constant	−0.19 (0.42)	0.03 (0.54)
AIC	35419.51	35424.88
Log Likelihood	-17697.76	-17697.44
Num. obs.	6768	6768

$^{***}p < 0.001$, $^{**}p < 0.01$, $^{*}p < 0.05$

Table S20: **Tobit regression analysis of the interaction between punishment scenario and first-degree alter's contribution two rounds later for the public goods game.** Tobit regression models of alter's contribution on ego contribution two rounds later interacted with low-cost, high-impact punishment condition (Model A) or interacted with all punishment scenarios separately (Model B), controlling for ego's and alter's contributions in two rounds previous, fixed effects for round and for cost and impact of punishment, using Huber-White sandwich errors to account for multiple observations of egos and alters. Data in these models are the pooled data across the five experimental procedures (no punishment, low-cost and low-impact punishment, low-cost and high-impact punishment, high-cost and low-impact punishment, high-cost and high-impact punishment).

	DV: Ego contribution in round t	
	Model A	Model B
Alter's contribution t-3	0.07 (0.03)*	0.13 (0.06)*
Ego's contribution t-3	0.61 (0.03)***	0.61 (0.03)***
Low-cost, high-impact punishment	5.76 (0.69)***	6.25 (0.85)***
High-cost, high-impact punishment	2.67 (0.48)***	3.12 (0.85)***
High-cost, low-impact punishment	−0.25 (0.49)	0.27 (0.83)
Low-cost, low-impact punishment	0.50 (0.49)	1.43 (0.86)
Alter's contribution t-3× Low-cost, high-impact punishment	−0.03 (0.05)	−0.09 (0.08)
Alter's contribution t-3× High-cost, high-impact punishment		−0.06 (0.08)
Alter's contribution t-3× High-cost, low-impact punishment		−0.07 (0.09)
Alter's contribution t-3× Low-cost, low-impact punishment		−0.11 (0.08)
Round 3	−0.59 (0.33)	−0.59 (0.33)
Round 4	−0.57 (0.34)	−0.57 (0.34)
Constant	−0.88 (0.53)	−1.36 (0.71)
AIC	26349.63	26353.31
Log Likelihood	-13163.82	-13162.66
Num. obs.	5076	5076

$^{***}p < 0.001$, $^{**}p < 0.01$, $^{*}p < 0.05$

Table S21: **Tobit regression analysis of the interaction between punishment scenario and first-degree alter's contribution three rounds later for the public goods game.** Tobit regression models of alter's contribution on ego contribution three rounds later interacted with low-cost, high-impact punishment condition (Model A) or interacted with all punishment scenarios separately (Model B), controlling for ego's and alter's contributions in three rounds previous, fixed effects for round and for cost and impact of punishment, using Huber-White sandwich errors to account for multiple observations of egos and alters. Data in these models are the pooled data across the five experimental procedures (no punishment, low-cost and low-impact punishment, low-cost and high-impact punishment, high-cost and low-impact punishment, high-cost and high-impact punishment).

	DV: Ego contribution in round t	
	Model A	Model B
Alter's contribution t-4	0.03 (0.04)	0.04 (0.08)
Ego's contribution t-4	0.50 (0.04)***	0.50 (0.04)***
Low-cost, high-impact punishment	5.79 (0.87)***	5.89 (1.06)***
High-cost, high-impact punishment	3.24 (0.63)***	2.60 (1.11)*
High-cost, low-impact punishment	−0.22 (0.64)	0.44 (1.08)
Low-cost, low-impact punishment	0.92 (0.62)	1.24 (1.07)
Alter's contribution t-4× Low-cost, high-impact punishment	0.14 (0.07)*	0.13 (0.10)
Alter's contribution t-4× High-cost, high-impact punishment		0.07 (0.11)
Alter's contribution t-4× High-cost, low-impact punishment		−0.08 (0.11)
Alter's contribution t-4× Low-cost, low-impact punishment		−0.04 (0.10)
Round 3	−0.11 (0.35)	−0.12 (0.35)
Constant	−0.97 (0.67)	−1.06 (0.89)
AIC	17431.95	17435.27
Log Likelihood	-8705.98	-8704.64
Num. obs.	3384	3384

$^{***}p < 0.001$, $^{**}p < 0.01$, $^{*}p < 0.05$

Table S22: **Tobit regression analysis of the interaction between punishment scenario and first-degree alter's contribution four rounds later for the public goods game.** Tobit regression models of alter's contribution on ego contribution four rounds later interacted with low-cost, high-impact punishment condition (Model A) or interacted with all punishment scenarios separately (Model B), controlling for ego's and alter's contributions in four rounds previous, fixed effects for round and for cost and impact of punishment, using Huber-White sandwich errors to account for multiple observations of egos and alters. Data in these models are the pooled data across the five experimental procedures (no punishment, low-cost and low-impact punishment, low-cost and high-impact punishment, high-cost and low-impact punishment, high-cost and high-impact punishment).

	DV: Ego contribution in round t	
	Model A	Model B
Alter's contribution t-5	−0.02 (0.06)	0.08 (0.13)
Ego's contribution t-5	0.43 (0.06)***	0.44 (0.06)***
Low-cost, high-impact punishment	5.95 (1.40)***	6.79 (1.73)***
High-cost, high-impact punishment	2.52 (0.93)**	3.27 (1.75)
High-cost, low-impact punishment	−1.16 (0.98)	1.02 (1.78)
Low-cost, low-impact punishment	0.48 (0.94)	0.94 (1.73)
Alter's contribution t-5× Low-cost, high-impact punishment	0.24 (0.11)*	0.14 (0.16)
Alter's contribution t-5× High-cost, high-impact punishment		−0.09 (0.17)
Alter's contribution t-5× High-cost, low-impact punishment		−0.26 (0.18)
Alter's contribution t-5× Low-cost, low-impact punishment		−0.06 (0.16)
Constant	−0.10 (0.98)	−0.98 (1.40)
AIC	8585.89	8589.02
Log Likelihood	-4283.95	-4282.51
Num. obs.	1692	1692

$^{***}p < 0.001$, $^{**}p < 0.01$, $^{*}p < 0.05$

Table S23: **Tobit regression analysis of the interaction between punishment scenario and first-degree alter's contribution five rounds later for the public goods game.** Tobit regression models of alter's contribution on ego contribution five rounds later interacted with low-cost, high-impact punishment condition (Model A) or interacted with all punishment scenarios separately (Model B), controlling for ego's and alter's contributions in five rounds previous, fixed effects for round and for cost and impact of punishment, using Huber-White sandwich errors to account for multiple observations of egos and alters. Data in these models are the pooled data across the five experimental procedures (no punishment, low-cost and low-impact punishment, low-cost and high-impact punishment, high-cost and low-impact punishment, high-cost and high-impact punishment).

References

[ER08] Martin Egas and Arno Riedl. The economics of altruistic punishment and the maintenance of cooperation. *Proceedings of the Royal Society B*, 275(1637):871–878, 2008.

[FC10] James H. Fowler and Nicholas A. Christakis. Cooperative behavior cascades in human social networks. *Proceedings of the National Academy of Sciences*, 107(12):5334–5338, 2010.
